# Sense of Coherence in Managers during COVID-19 and the New World of Work: A Mixed-Method Study

**DOI:** 10.3390/ijerph182111492

**Published:** 2021-10-31

**Authors:** Claude-Hélène Mayer, Cemonn Wegerle, Rudolf M. Oosthuizen

**Affiliations:** 1 Department of Industrial Psychology and People Management, College of Business and Economics, University of Johannesburg, Johannesburg 2006, South Africa; cemonnwegerle@gmail.com; 2 Department of Industrial and Organisational Psychology, School of Management Sciences, College of Economic and Management Sciences, University of South Africa, Pretoria 0003, South Africa; Oosthrm@unisa.ac.za

**Keywords:** mixed-method study, salutogenesis, sense of coherence, managers, COVID-19

## Abstract

During COVID-19, the working world has changed inevitably, and many managers experience extreme strain and stress. This study determines how managers cope with the changes during COVID-19 from a positive psychology and salutogenic perspective. It employs a hermeneutical research design and an interpretivist paradigm by using a mixed-method research approach in which managers’ sense of coherence (SOC) is investigated quantitatively through the 29-item Life-Orientation scale and qualitatively through semi-structured interviews. Purposeful and snowball sampling techniques are used. The sample consists of 17 managers. Data were collected in different organizations within South Africa and analysed through content analysis, linking quantitative and qualitative data in a holistic, integrated and complex way. In terms of the quantitative findings, the managers scored at the medium and higher end of the SOC-scale in comprehensibility, followed by manageability and finally meaningfulness. Male managers in the age group 47–57 scored highest. Female and younger managers scored lower on average. Lowest scores in comprehensibility and manageability were scored by a young female manager, while in meaningfulness the oldest male participant scored lowest. The qualitative findings show that high scoring SOC managers apply complex thoughts to the present and future workplace scenario. Individuals with lower SOC scores do not present as much knowledge, complex thinking and argumentation structures during the interview in comprehensibility scores as high scoring SOC managers, yet still acquire resources to manage the workplace (manageability). High meaningfulness scores are associated with creating meaningful workplace interaction (human–human and machine–human), knowledge distribution through technology, impactfulness, experiencing the job as meaningful, including helping others, and achievements. Managers have a complex view of the world and findings show the complex connections of a high/low SOC scores and the managers’ explorations and systemic understanding regarding their managerial world. Conclusions and recommendations for theory and practice are given.

## 1. Introduction

The COVID-19 pandemic has pushed employees globally to work in new ways, eliciting interventions such as social distancing, travel restrictions, virtual work, and new essential teamwork methods [1,2]. Such interventions sparked by the COVID-19 pandemic presented employee behaviour changes, which might impact employees’ and managers’ psychological, rational and somatic well-being, and thereby their performance [3]. The immersion of COVID-19 activated organisations to contain the impact on performance of employees [4] and become aware of stress, insufficient infrastructure, missing work milieu and virtual colleagues, unrealistic performance expectations, impaired manager-employee relationships, and difficulty establishing trust in virtual work environments [3].

Research also indicated that organisations providing an improved work life balance through virtual work options pave way for a more productive workforce as employees feel more motivated [4]. Graves and Karabayeva [3] stated that virtual work provides employees with flexibility in work and increased availability of time due to the absence of commuting. More importantly, it improved the access to better talent around the globe, which might increase the average individual’s performance [5,6]. However,, besides the advantages of the new world of work, stress levels of employees have risen during COVID-19, and it has been pointed out that resilience—the ability to recover after a stressful period [7]—is needed, since higher levels of resilience amongst employees reduce the harmful effects of a crisis [8]. Strengthening resilience [9,10] can be important for crisis management. Managerial recommendations for strengthening resilience include accepting the inevitable; focusing on positive gains; drawing attention to organisational growth; measuring and attending to employee’s day-to-day emotional states and well-being and improvements in employee health; taking responsibility; understanding organisational limitations; reversing negative thoughts; and knowing organisational strengths.

The Sense of Coherence (SOC) is a competence that supports managers perceive the situation as understandable, manageable, and meaningful, facilitating the activation of their resilience [11]. The SOC, proposed by Antonovsky [12] within the salutogenic framework, explains why certain managers do not lose their wellbeing when faced with demanding situations, reinforcing personal reserves to prevent both mental and physical disorders [13]. The SOC is a foundation of health advancement and a good predictor of burnout, depression, and job satisfaction rates [14,15]. A strong SOC can support mental and bodily health and well-being, also in the workplace, as well as complex understanding, high levels of manageability and a strong sense of meaningfulness [16].

This research focused on exploring the SOC during the experience of COVID-19 in the workplace through quantitative and qualitative data collection. The aim of this study was to explore the SOC in managers across different organisations in South Africa during COVID-19, aiming at understanding the SOC levels of managers in-depth and holistically and their sub-components of comprehensibility, manageability and meaningfulness in the context of their present work situation and in connection with the future of work.

## 2. Managing during COVID-19

Managing employees and organizations during COVID-19 has changed dramatically [1,2,3,4]. Research has stated six important factors for managing during COVID-19 [17]:

Researches advocate that managerial resistance to virtual innovation in the workplace is a key challenge in its effective practice. The effective and productive management of virtual employees demands a result-based management approach. This approach entails ascertaining goals distinctly, determining functions to be accomplished, and monitoring, evaluating and deliberating progress without overly arduous reporting obligations [18,19].

Appropriate equipment and training for managers and virtual workers includes possessing and having access to computers, laptops and apps/software for virtual work, adequate and timely technical support, and capacity building in the area of training for both managers and virtual workers. Since the possibility and risk of social isolation associated with virtual work exists, virtual workers need to be assisted to stay connected with colleagues, technical teams, supervisors and the organisation as a whole [20].

Defining clear expectations in virtual work arrangements is vital to monitor and assess actual performance from expectations. All parties involved need to be certain about expected results to be accomplished by virtual workers, hours for contact and number of hours of engagement, conditions of employment, ways of evaluating progress and reporting results. There must be clear rules and regulations to guide managers and virtual workers [18].

Time autonomy for virtual workers offers employees flexible time usage. This flexibility is important if virtual work is to be made successful. Flexibility in time usage allows virtual workers to align their paid work with personal responsibilities, such as taking care of their children and other family commitments [21].

A boundary management strategy enables virtual workers to have a dedicated workspace free from distractions, and the ability and freedom to disconnect from work at specified times devoted for rest and personal life. 

Trust is often regarded as the “glue” that holds other factors together. Trust between managers, virtual workers, and their colleagues is fundamental to be effective [17]. Trust in institutions (i.e., perceptions of them as competent, honest and benevolent [22,23], helps people manage complexity and is crucial for legitimising decisions made by managers [24,25,26]. Therefore, actions and communication should aim to maintain or increase trust [27].

In connection with consolidating positive organisational norms, emphasising the existence of a shared endeavour and organisational solidarity, a shared appreciation of interdependence amongst employees in an organisation, and recognising that stringent policies are useful in the context of high organisational dangers [28], can be useful during organisational emergencies that require collective action [29]. Increasing an employee’s sense of organisational empathy towards those at utmost risk could be helpful in the context of the COVID-19 change phase for promoting pro-organisational actions.

An organisational response to fear caused by earlier inconceivable hardship is to attempt to control the fear by denying disturbing information and taking actions that are not consistent with employee or organisational interests [30]. Hardship can be alleviated by emphasising self-efficacy (the belief that an action can be completed [31]) and response efficacy (the belief that an action can reduce a threat [32]). Managerial explanations on what should be done, and the reasons for doing it, can promote response efficacy, as well as an increase in comprehensibility, manageability and meaningfulness, making transitions as easy as possible so that employees understand the actions they should take to protect themselves. Providing feedback on these actions can increase self-efficacy [33] and contribute to the three components of SOC.

## 3. Salutogenesis and Sense of Coherence

The health crisis triggered by COVID-19 and the preventive measures taken to gain control have caused a strong psychological impact on managers: Risk exposure, uncertainty about how to approach the disease, care and emotional overburden, lack of resources, or unclear ever-changing protocols are, among others, psychological distress risk factors for managers who have faced this dramatic scenario [34].

Coping in this kind of crisis has been positively related to SOC, and both SOC and engagement have been negatively related to exhaustion [35]. It is known that SOC has a negative correlation with developing post-traumatic stress, which could be equal to the stress produced by the COVID-19 pandemic, though this correlation is positive with regards to tolerance to extraversion and frustration [36]. A manager’s ability to perceive a stressful situation as understandable, manageable, and meaningful, allowing them to use their reserves to effectively cope with it [37]. SOC influences the improvement and continuation of health; the higher SOC, the worthier the manager perceives his/her health, in particular mental health [38]. It has been described as a factor of wellbeing and a shielding factor against psychological distress and overload [34,37], as well as a component that reinforces personal resilience [38]. In contrast, low levels of SOC have been associated with burnout and depression [39]. SOC relates to workplace flexibility, job satisfaction, and illness-absenteeism [40], as well as being a shielding factor against stressors that come from the work context and complexities regarding work-life balance [39]. After the first months of the COVID-19 pandemic, topical research has described psychological distress amongst managers [41,42,43,44]. However, SOC, the associated risk factors, and its potential as a protective measure against distress are less explored.

Recent research focused on SOC as the contrivance underpinning the stress-health link by exploring it as a moderator. However, while SOC has constantly indicated positive direct links with health, its moderating function in the stress-health relationship needs further explanation [45,46,47]. Certainly, the power of SOC to cushion the negative effects of stressful experiences on health might depend on the type and seriousness of stressful events [48]. It is evident that SOC may be a formidable protective factor to reduce stress imposed by the COVID-19 outbreak and promote well-being. Schäfer et al. [49] posited that SOC predicted changes in psychopathological symptoms from COVID-19 pre-outbreak (at the end of February) to post-outbreak (1 month later). Findings indicated that a significant proportion of the sample experienced mental health problems related to the COVID-19 pandemic (especially among women and younger participants), but higher pre-outbreak levels of SOC were related to smaller clinically relevant changes in psychopathology (for example increases or decreases) [49]. Thus, higher levels of SOC buffered the impact of COVID-19 stressors on general health but did not result systematically in lower symptom levels.

## 4. Research Methodology

The mixed-method research methodology used in this study will be explained in the following.

### 4.1. Research Design, Paradigm and Strategy

This study uses a mixed method research design within the phenomenological (hermeneutical) research paradigm [50], using interpretative hermeneutics [51,52]. The research design is a mixed-method design since it uses qualitative, as well as quantitative methods (an interview structure and a questionnaire). The paradigm, however, is subjectivist, constructivist and interpretative in nature since the analysis and the interpretation of the mixed methods is conducted from a qualitative perspective only [50]. The research aims at understanding the phenomena from a holistic and complex perspective through the lens of the combined approach of the phenomenological and hermeneutical perspective, thereby attributing meaning in context [53] to create “Verstehen” (understanding) [54]. The research strategy applied is abductive in nature [55], using a mixed qualitative and quantitative methods method approach for responding to the research questions. 

The following Table 1 shows the research process.

### 4.2. Data Collection and Analysis

This research applied one quantitative questionnaire (29-SOC-Life-orientation) [12] and qualitative methods (in-depth interviews) to collect, analyse and interpret data. The collection, analysis and interpretation of the quantitative data supplement the analysis and interpretation of the qualitative data within the phenomenological research paradigm through the use of descriptive statistics. The SOC-questionnaire was measured in each manager, and interviews were conducted with all of them. The interviews contained questions, e.g., about the understanding of the world of work and mental health, the resources to manage mental health and meaning in life. They were then connected and explained by referring to the qualitative data analysis. Completing the questionnaires took approximately 10 min and confidentiality and anonymity of the data were assured. Data collection methods included the following analyses of documents and secondary literature; questionnaire survey; and in-depth interviews. 

The SOC-questionnaire consists of a 29-item scale to measure individual SOC. Each single item offers seven possible answers [13]. The respondent is requested to select the number which best corresponds to the extent to which each statement is applicable to him/her. The numbers, ranging from 1 to 7 on a 7-point Likert scale, represents extremes at each end of the scale [16]. The questionnaire measures the individual’s enduring tendency to see his/her life space as more or less ordered, predictable and manageable. Altogether, 13 of the items (1, 4, 5, 6, 7, 11, 13, 14, 16, 20, 23, 25 and 27) are formulated “negatively” and need to be reversed in scoring [56]. High scores, therefore, express a strong SOC. The following scores are determined by adding the values assigned to the items indicated comprehensibility (1, 3, 5, 10, 12, 15, 17, 19, 21, 24 and 26), manageability (2, 6, 9, 13, 18, 20, 23, 25, 27 and 29), and meaningfulness (4, 7, 8, 11, 14, 16, 18 and 22). A high score represents a strong SOC. Such an individual, as opposed to the individual with a weak SOC, will be able to comprehend, manage and engage with the nature and dimensions of a stressor as one to which the individual need not succumb [57]. The SOC Scale yields internal reliability indices of between 0.78 and 0.93 and test-retest reliability between 0.56 and 0.96 [58] which has been confirmed through various research (e.g., [59]. Construct validity varies between 0.38 and 0.72 [60] and is acceptable [61].

For the analysis of the questionnaire, only descriptive statistics were used, since the results of the questionnaires were only used to explore the SOC scores matching them with the qualitative findings and the interpretation of findings in the context of the SOC questionnaire scores, as previously conducted [16]. That means that the authors analysed the findings based on the Likert scale by using the usual descriptive analysis techniques as indicated above with regard to the scoring and reverse scoring processes. The qualitative data were analysed through thematic analysis and the process of identifying patterns in meaning across the data [62,63]. Quantitative and qualitative data were analysed through inductive processes as well as deductive processes, leading to an abductive research approach [64]. The back and forth of abductive processes help to explore the best interpretations of the data to construct meaning [65] by interpreting the quantitative and qualitative set of data.

### 4.3. Sampling and Sample

The sampling methods used were purposive and snowball sampling [66,67] to include managers working in organizations in South Africa and who are over the age of 18 years old. Sampling criteria included being on a managerial level and speaking English fluently. Mainly white individuals responded to the research request and agreed to participate in the research voluntarily. Altogether 17 managers participated in the study, displaying the biographical information provided in Table 2.

### 4.4. Quality Criteria in Mixed Method Research

The study is anchored in the mixed method research paradigm, however, the findings are analysed, interpreted and discussed from a qualitative perspective, and anchored within qualitative quality criteria. Trustworthiness is established through by ensuring credibility, transferability, dependability, and conformability [68,69].

In this research, credibility is pursued by presenting the study in transparent ways, as well as referring to the research aims [70]. Additionally, dependability is shown through the reliability of the findings and the consistency presented in the findings and the transparent presentation of the research process. Since the process of the research study is displayed in detail, transferability was achieved [71]. Conformity was reached through the discourses on the findings with other researchers [72].

### 4.5. Ethical Approval

Ethical approval for this research was given by the University of Johannesburg, in Johannesburg, South Africa.

Before the participants agreed to participate in this research, they received all the information on the study, its aims and processes. They could make a decision to participate or not. Participation was therefore voluntary, and they all had the right to withdraw from the study at any point in time. Further, they could exercise the right of informed consent [73]. The researchers guaranteed participants’ anonymity, confidentiality and precautions were put in place to prohibit the disclosure of personal information to other parties. To avoid deception, reporting was conducted in a transparent and honest manner [74].

### 4.6. Limitation of the Study

As in any study, there have been limitations. It is limited to the focus of the theories, the research methodology, including the sample and its bias with regard to the South African organizational context and the majority of participants belonging to the white cultural groups within South Africa. The study is further limited to the relatively small sample size and the quantitative data are only analysed and evaluated from a qualitative and descriptive perspective. Although the data have gone through an intersubjective validation process of the researchers, the analysis might be biased by the subjective view of the researchers which are anchored in their intersectionalities, including social group belonging, cultural background, gender and age. The data can in no way be generalized, but rather provide in-depth information on SOC and the interrelationship of the quantitative and qualitative data.

## 5. Findings

The SOC findings presented are quantitative and qualitative in nature and presented combined to provide a holistic insight.

### 5.1. Comprehensibility

**Figure 1:**
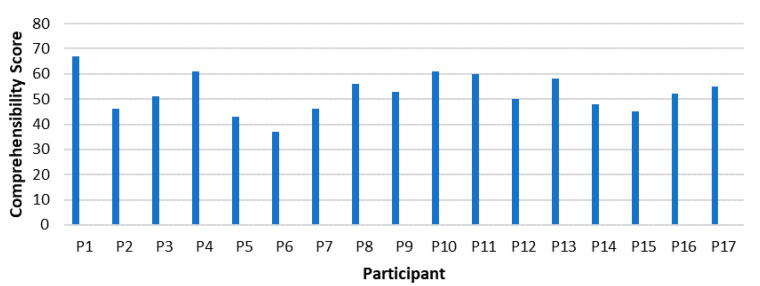
Comprehensibility in Managers.

Figure 1 demonstrates the scores achieved by each participant on the comprehensibility sub-component of the SOC-29 Questionnaire.

Figure 1 illustrates that P1 (67) scored the highest on the comprehensibility sub-scale, followed by P10 (61), P4 and P11 (60 both). whereas P6 (37) scored the lowest. All high scoring participants are male, and all are 47 to 57 years old, meaning that they are in their mid-or late career stage. P6 is a female manager in her 30′s. Findings show that younger women score the slowest in comprehensibility throughout the comprehensibility scores, while middle and higher aged males score the highest in comprehensibility. It might be interpreted that elder men in managerial positions are settled, know their business well, have experienced consistency and are aware of what they are doing. They might also have a good insight into the world of work and might be easily able to anticipate future developments. Through consistent work experiences, good networks and (male) support systems in their business, they might have a good insight and comprehensibility. Women, contrarily, in particularly younger managers, seem to be challenged with comprehensibility. Middle and elder women display higher scores in comprehensibility, but also not as high as their male counterparts. 1: The numbers in brackets are the SOC-scores on the scales.

The participants were asked how they understand their workplaces and the workplace changes in terms of their expectations for the future. Most of the participants display a good or high understanding of their workplaces in present and also with regard to a predictable future. The topics that were mainly discussed with regard to the workplace were: (1) workplace and job changes; (2) increasing use of technologies; (3) remote work experiences, and (4) health and well-being.

#### 5.1.1. Workplace and Job Changes

In the business environment, seven participants noted that the workplaces are driven by rapid changes and that organizations and employees need to anticipate the change and go with the flow.

*“…I see it as obviously uhm… a change in the business environment* *…” (Participant 1, Male),*

Whereas two participants averred that it was a necessity in the workplace:

*“I think if if [sic] the workplace doesn’t embrace it and change in order to use it to its own benefit; I think … companies won’t survive.” (Participant 2, Male).*

The majority of participants thought that their job descriptions would not change in the near future, but the way the work would be carried out would be. They also anticipated getting additional tasks in the job descriptions. However, selected managers indicated that a few jobs could be automated.

*“Uhm I… don’t… think my job description will change as much in the near future, uhm we will still have projects, we still need to deliver on certain time…” (Participant 6, Female),*

Participants were strongly aware of the anticipated workplace changes, as well as on workplace aspects which might not change and rather stay the same.

Additionally, three participants believed that their jobs could be automated:

*“However, going back to the jobs that I had previously and managing teams, curriculating, uhm and course design can be absolutely done by automated technology, because it can reach further the data gathering process, the comparison process between what all other institutions are doing, I think that can be done better by an automated service.” (Participant 12, Female).*

Five participants expected that in future employees will lose their jobs:

*“…I think it’s not good in terms of jobs. Uhm, you know, because, you know, computers are essentially taking over where people used to do these things.” (Participant 8, Male),*

And four participants expected new jobs across the industries:

*“…look at, it it [sic] creates new job opportunities for people that… uhm, it creates new work. We don’t even know what kind of jobs are [sic] going to be out there…” (Participant 10, Male).*

This participant highlights the new opportunities coming in post-COVID-19 times and also emphasizes that there are uncertainties what exactly the future will bring and what will be needed in future workplaces arrangements.

#### 5.1.2. Increasing Use of Technologies

Participants further felt that in future, through the increasing use of technology, their work would become easier and more efficient. They were of the opinion that the virtual workplace would become more prominent, and that they would most probably experience a decrease in personal contact with their colleagues and other employees. They also anticipated that, in the future world of work, certain jobs would become redundant jobs and new industries and jobs would be created.

*“And my my [sic] job probably would not be as easy if it wasn’t for technology. So… I think… it is made much of a lot easier.” (Participant 3, Male).*

Additionally, coding and data analytics in the work environment are becoming more prominent:

*“…data analytics is the way forward if you can if you can [sic] code, you’re probably going to be rich, and had [sic] lots of jobs…” (Participant 3, Male).*

This participant is highly aware that skills and knowledge in certain areas and industries will contribute to the individual’s advancement and the opportunities a person will have within the workplace. Knowledge of new technologies is obviously also associated with power and wealth and the choice of jobs and workplaces.

#### 5.1.3. Remote Work Experiences

Nine participants mentioned that remote work would become more acceptable in the world of work:

*“…we’ve already seen the changes, uhm we’ve discovered that we can work remotely.” (Participant 7, Female).*

Nine participants, predominantly women managers, voiced their concern about the decline in human interaction in the workplace:

*“…in the human context, we are more and more reliant on technology and more more [sic] isolated from other human beings, that personal contact is falling away, and it’s being replaced by technology…” (Participant 5, Female),*

and

*“I think, obviously, losing that personal touch in the work environment might be a problem.” (Participant 11, Male).*

Remote working experiences are a point of discourse during the interviews. Participants emphasise that people get used to working remotely. At the same time, they are aware that technology increases in impact on human interaction and one participant even mentions that human touch is being lost in workplace interactions, which might be viewed as negative.

#### 5.1.4. Health and Well-Being

The managers further revealed their belief that the changes in the workplace had a good impact on their health due to new technologies and the developments in the medical field. However, a few participants felt negatively affected negatively due to longer working hours, less exercise and fatigue. A few participants did not experience any direct effect on their health.

Eight participants revealed that their life and work is nowadays easier and more comfortable:

*“It’s made my life easier. I get here, I didn’t have I didn’t have [sic] a computer when I started here, or my other companies where I worked, I had a computer to keep in touch with uhm, people overseas or contacts…” (Participant 14, Male).*

Furthermore, seven participants stated that the technology assisted to monitor exercise and made it easier to be motivated to exercise:

*“Uhm, and it also motivates you, if you see where you at with it.” (Participant 11, Male).*

In addition, six participants said that the technological impact on workplaces and life in the health sector make a huge difference in terms of health and well-being:

*“Oh, health wise, if I were to get the knee replacement, I can get a 3D printed knee…” (Participant 12, Female),*

and

*“…if you look at the technology in in [sic] the medical fraternity, if you want to call that—how that’s going to decision making—all those nanotechnologies in in [sic]… operation stuff, I think that will have an impact.“ (Participant 10, Male).*

Exploring the quantitative findings in the context of the qualitative generated findings, the high comprehensibility of P1 is indicated by how the participant describes his understanding of the situation and how he anticipates the future in terms of opportunities and challenges. P6, as the lowest scoring person in comprehensibility also showed a lack of understanding the processes of the business and the impact of the rapid changes in the workplaces. She did not give detailed descriptions and did not anticipate the impact of change in her workplace.

With regards to the entire sample, the overall score of the sample on the comprehensibility component falls within four and five on the Likert scale, which indicates that the comprehensibility scores are between the medium and higher scores, but not as high as could have been expected.

### 5.2. Manageability

The findings indicated that the participants used various resources at their disposal to cope with change and stay abreast of the new developments. Managers use the following to manage their tasks: 1) Skills and knowledge to manage; and 2) Managing resources.

**Figure 2:**
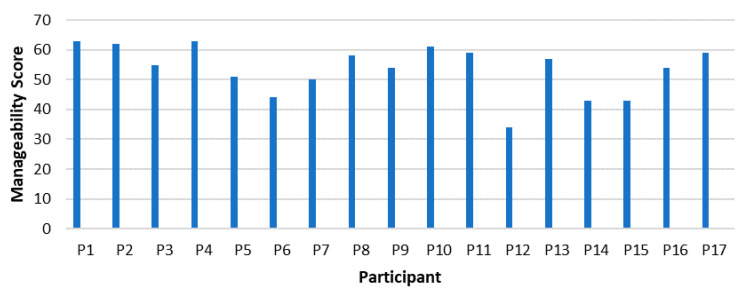
Manageability in managers.

In the following, Figure 2 demonstrates the scores achieved by each participant on the manageability sub-component of the SOC-29 Questionnaire.

In Figure 2, P1 (63) and P4 (63) scored the highest on the manageability subscale, followed by P2 (62) and P10 (61). With regard to comprehensibility, the individuals who are scoring highest in manageability are male managers between 47 and 57 years. Again, these are the male managers managing Engineering and IT–areas which are leading areas at present and predictably in the future of work.

P12 (34), a female manager in her thirties again scored the lowest on this sub-scale of manageability, as with regard to the component of comprehensibility.

It might be interpreted that the male middle and higher aged managers score relatively high because of their experience of consistent ability to manage their jobs and their tasks. They are confident that they can manage the workplace and have enough advanced skills to even drive the future of work, especially with their knowledge base in Engineering and IT.

#### 5.2.1. Skills and Knowledge to Manage

Twelve participants stated that continuous learning was an essential activity to manage present and future work:

*“Learning as you go, I mean, it’s impossible to get all the information in one shot…” (Participant 3, Female),*

Furthermore, twelve participants admitted to sharing knowledge and skills with others who were knowledgeable was a key practice to stay abreast with new developments:

*“And other thing as well is connecting with people face to face, virtually, and learning from their experiences and then determining, you know, what is, you know, how can I respond to that, to ensure that they have uhm, a good experience.” (Participant 6, Female).*

Additionally, twelve participants stated that they used their current skills and knowledge to cope and manage the changes presented:

*“Uhm, but I [sic] think for me, skills that is important still would be active listening, making sure I listen to what people say, that connecting thing that I talked about earlier…” (Participant 2, Male),*

*“…then obviously analyzing skills, ja ja [sic], to analyse all this information that you get, because it’s going to be an information overload and you need to to [sic] know how to interpret that information.” (Participant 11, Male),*

and

*“…I mean, there’s there’s [sic] a technical answer, and that and that [sic] is remain—understanding the cloud technologies out there in computing, and what kind of solutions exist.” (Participant 10, Male).*

Nine participants said that staying informed about the new developments was a coping mechanism:

*“…if we think about and inform ourselves, we will be able to take advantage of of [sic] opportunities that present themselves.” (Participant 1, Male).*

Nine participants revealed that they had upskilled themselves through training courses and internet platforms to remain relevant:

*“…we’ve had quit a few platforms we had to because this company will use Teams, that company will change software flatforms that are being used, way of training is changing.” (Participant 5, Female),*

and

*“…because we are in a tech world, technology change often. So, we do many course change [sic], we do a lot of courses and stay up to date with new technology all the time.” (Participant 6, Female).*

The findings further indicated that six participants believed that adaptability was important:

*“Uhm so ja, I think just basically people that are stuck in their ways are not going to cope [laugh] they will have to be adaptable.” (Participant 2, Male)*.

Several of the employees refer to the idea that skills and knowledge need to be managed and that people need to increase their flexibility and adaptability. Further rapid change is expected and participants do not see any other solution than to adjust and adapt.

#### 5.2.2. Managing Resources

The findings also indicated that certain participants used software as well as technological and financial resources to manage their workplace changes:

*“So, I would have an audible subscription and listen to all the latest books on technology, leadership, people development. So uhm I think money helps obviously, to enable these things, I’d tap into that to make sure that my learning continues.” (Participant 10, Male).*

In addition, one participant used exercise to clear his head:

*“And then my biggest coping mechanism is my training. Uhm, I [sic] just do it to get out or to get away or to clear my head.” (Participant 4, Male).*

The findings also showed that managing time on devices and exercising were necessary to have a work-life balance and to cope with negative workplace aspects. Six participants asserted that it was important to manage their electronic devices:

*“Uhm, on a personal level, on my phone, I have been known to have some screen time limiting apps, uhm, just to sort of manage the amount of time I spend in front of my phone, uhm, there isn’t really much I can do about the need to be in front of a computer at work.” (Participant 15, Female).*

Integrating the findings from the SOC questionnaires and the interviews, P1 clearly describes the means by managing the change brought through learning, identifying the risk and opportunities presented. He displays an in-depth ability to manage his resources and thus his workplace. In addition, P4 also mentions that research and gaining knowledge is important while considering their impact limitations. All of the high scoring managers are aware of their abilities and resources and use them well. P12 scored the lowest in the manageability score; however, the participant describes her coping mechanism as upskilling and learning. She might not feel that she is coping well, but she has put measures in place to increase her manageability. Therefore, the manageability seems to be work in progress.

While looking at the sample, the overall score of the sample falls within the medium to the higher end of the Likert scale, which demonstrates that the sample attained a high manageability score.

### 5.3. Meaningfulness

Overall, the participants’ view of the workplace as positive and optimistic; however, some are critical. In addition, the meaningfulness scores showed that the participants viewed the workplace challenges and changes as meaningful and partly as a worthwhile effort. During the interviews they mainly related the meaningfulness in their life to their career.

**Figure 3:**
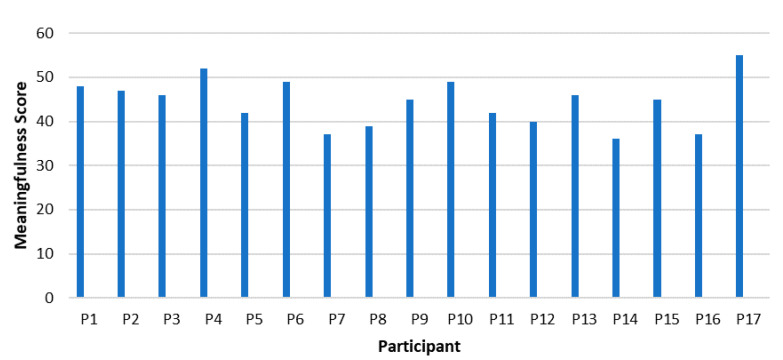
Meaningfulness in Managers.

Figure 3 illustrates that P17 (55) scored the highest on the meaningfulness sub-scale, followed by P4 (52); P10 and P6 (49 both), whereas P14 (36) scored the lowest in this sub-scale. Again, the highest scores are reached by white male Afrikaans-speaking white participants aged 47 to 51. The lowest score was also reached by a white male Afrikaans-speaking 63-year-old participant who is an Executive Director of an old age home and the oldest participant. The female participants score on average lower than the male participants with exception of P6 who is the only high scoring female manager (white Afrikaans-speaking).

Participants indicate that their jobs are meaningful due to their fact that they can help others, e.g., for distributing knowledge to more people than without technology and thereby having an impact. Although some participants experience their jobs as such as meaningful, others make their work meaningful only through their achievements.

#### Meaningfulness in the Workplace

Eight participants asserted that their job is meaningful when they impacted other people’s lives by reaching more people using technology and developing their knowledge and skills:

*“…I can reach more people through technology that would be great, uhm that I could get the knowledge out there. Uhm, you know, get more knowledge to more people.” (Participant 5, Female),*

and

*“…it’s just leading people really, whatever, if it’s technology or whatever, it’s literally being part of somebody’s growth. Uhm, luckily, in my space, I have that opportunity and I’ve got the financial backing of my company to actually uhm, grow people in their careers without any limitations, you know…” (Participant 10, Male).*

Furthermore, two participants stated that their jobs would be more meaningful if services that assisted humanity such as more access to psychological services were provided:

*“So, I think what would make my job meaningful in… is… more access to to [sic] psychological services or to intervention… If you move up virtual, you can pre-record a lot of the content and people can listen to it and then just have half the session with the person thereby therefore doubling the amount of people you can reach…” (Participant 3, Male).*

Four participants mentioned that the future of work changes make their work meaningful in terms of providing solutions for major challenges, such as water problems, reducing human error, providing important information and acquiring a battery pack to be outdoors more:

*“…the fourth industrial revolution is one of the tools that you will have to obviously take into account.” (Participant 1, Male)*

*“If a computer can tell me I am going to be done on this day, and he will be done by that day because he is a computer that will make my life meaningful, because then I don’t have a human interaction or dependent on any human failures or faults.” (Participant 6, Female)*

Four participants found their jobs meaningful when they had personal contact with people:

*“To connect with people and make sure that you have a positive impact. I think the fourth industrial revolution… uhm, can assist me in that, I mean, if I use it the right—in the right way.” (Participant 2, Male).*

Two participants stated that their jobs they would find their job meaningful if they were challenged more often and did not rely on technology: *“…I like being challenged. So, I almost don’t want to use the technology, I want to use my brain.”* (Participant 8, Female). 

One participant opined that a sense of achievement would make his job meaningful:

*“And does your professional life at least give you a sense of achievement and, and and [sic] that I do have at the moment.” (Participant 1, Male).*

Two participants communicated that their jobs would be meaningful if they delivered good work at the end of the day and two participants said that their jobs would be meaningful if they were assured that the company was profitable and sustainable:

*“…to make sure that we are profitable, that you do make money so that the company will be sustainable.” (Participant 11, Male).*

Five participants asserted that they found their jobs meaningful, by saying:

*“Ja [sic], I would like to think that my job is meaningful at [sic] this stage…” (Participant 11, Male),*

Meaningfulness on the job is experienced through bringing solutions to major human challenges, human–technological interaction which create increased knowledge, human–human interaction which creates growth and brings psychological services, solving problems, having personal contact with people, having to solve challenges, achieving goals, creating a sustainable organization and having a job that is in itself meaningful.

P17 scored highest in meaningfulness and stated in the interview that the anticipated future of work had a negative impact on his work-life balance, but that he could find meaningfulness of the rapid changes in the work-life impacting positively on family health. P14, having scored lowest in meaningfulness, described the meaningfulness of work in terms of positive and negative consequences, but appeared to be critical of the meaningfulness of the radical workplace changes, which might explain the low scoring. He did not refer to the meaningfulness of workplace changes for him, but he felt that the changes bring an increase in knowledge, and greater access to information.

The overall score of the sample falls within the higher end of the Likert scale, which demonstrates that the sample attained a medium to high meaningfulness score.

### 5.4. Overall SOC Scores

**Figure 4:**
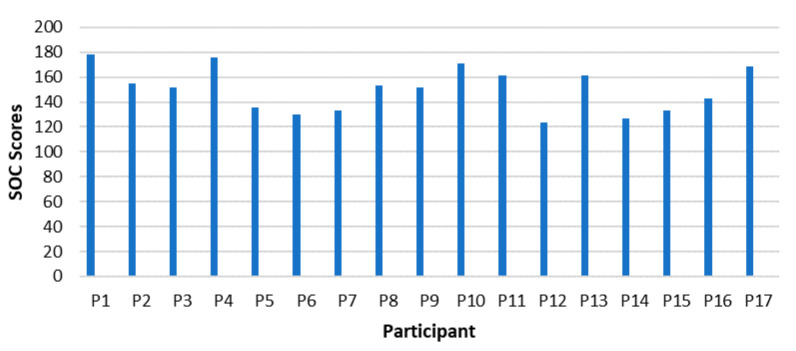
Total SOC Scores in Managers.

Figure 4 demonstrates the total SOC scores achieved by each participant.

Each participant’s SOC scores are illustrated, where P1 (178), P2 (155), P3 (152), P4 (176), P8 (153), P9 (152), P10 (171), P11 (161), P13 (161), and P17 (169), whereas P1 attained the highest overall SOC score. The lowest SOC score is 124, which was attained by P12. The sample’s SOC scores range from medium to high scores, which indicates that none of the SOC scores are weak.

The highest overall SOC scores were reached by male, Afrikaans-speaking managers in the age-group 47-57. Overall male managers scored higher than female managers: the eight highest SOC scores are maintained by male managers, the highest scores by female managers only come in at rank 9 and 10, scoring 15 and 16 points lower than the highest SOC score. The highest SOC scores by females are reached by English-speaking white female managers, the first aged 58 and the second aged 38 years.

## 6. Discussion

Interpreting the findings of this research study with regard to the literature, it can be highlighted that the managers score in the higher and medium range of SOC which means that they all of relatively high competences that supports managers perceive the situation of COVID-19 as understandable, manageable, and meaningful, facilitating the activation of their resilience [11]. Therefore, it might be assumed that managers participating in this research do not suffer to much during COVID-19 but have a good understanding of the situation and of what is happening. They are also, to a high degree able to manage the situation and cope with it. However, in comparison to the first two subscales of SOC they do not seem to have such a high, but rather a medium-based meaningfulness with regard to their jobs and careers. Strengthening resilience [9,10] in the context of SOC development in managers is important to create mental health and well-being. The SOC is a foundation of health advancement and a good predictor of burnout, depression, and job satisfaction rates [14,15] and this might be reflected in the findings, since the managers score relatively high in overall SOC scores.

Researchers stated important factors during COVID-19 [17,18,19] and it can be highlighted that in this research, the managers interviewed do not present managerial resistance to innovation. They also agree with the literature that appropriate equipment and training is needed to deal with the situation and the rapid changes [20]. Partly, they also fear collegial isolation and work towards mutual communication in the new world of work. They aim at good work relationship and human-human and human-machine interaction.

In the interviews, managers, however, do not speak about their expectations in new work relations to manage work [20]. They are very aware about the advantages of flexible work arrangements and adaptability [21], as is also mentioned in the recent literature. Managers are further aware of the importance of their boundary management and in terms of comprehensibility, but also with regard to manageability and coping, as mentioned in the literature [21]. Trust [17,22,23], as such, is not mentioned in the interviews, but might be part of the description of creating good human–human interactions. In connection with the positive human-human interactions, managers aim at making competent, complexity-related decisions [24,25,26]. Managing through informed communication and action is also important for the managers interviewed in the COVID-19 workplace situation, as described above [27].

Finally, managers in this study feel that they can have an impact as long as they are upskilled, informed, able to grasp the complexity of the situation, activate their resources and therefore complete their action, as described in the previous studies [31]. If this is the case, the managers feel also response efficacy, coping with the potential threat [32,33]. Several managers, and in particular female managers, feel that they need to upskill further and learn and gain knowledge about the situation [33], thereby increasing their comprehensibility and resource management and maybe even their meaningfulness. The research at hand agrees with the previously described six factors to manage times of COVID-19 well in the workplaces. This further points out that COVID-19 might trigger a strong (negative) psychological impact on employees and managers in the workplace; however, a strong SOC can prevent against burnout or psychological breakdown and exhaustion [35].

Since the managers at hand score relatively high in SOC, they display the ability to perceive a stressful situation as described as understandable, manageable, and meaningful, allowing themselves to use their reserves to effectively cope with it [37]. Generally, managers in this research seem to cope quite well with the pandemic [36] and maintain their mental health [38] and resilience [39]. Again, the findings in the dataset seem to support previous research in its full strengths. Further, the research shows that low scores in SOC are associated rather with negative views on the future and the challenges ahead than the positive views, as it is in the literature connected to frustration, burnout and depression [39]. High scoring managers also mention their preference of flexibility and their satisfaction with their job [40]. They also do not seem stressed by the situation and are able to balance the stressors with the management of their resources [39].

Recent research has emphasised the psychological distress of managers during the first months of COVID-19 [41,42,43,44]; however, these datasets indicate that the managers interviewed cope relatively well with the situation, assumingly because of the relatively high and medium SOC scores. All managers seem to have more or less resources and protective measures in place to deal with the potential distress. SOC seems to be health protective also in this sample, moderating stress levels [45,46,47,49]. Previous findings indicated that a significant proportion of the sample experienced mental health problems related to the COVID-19 pandemic (especially among women and younger participants) [49]—this might also be relevant to the sample in this research study. Women and younger participants might be at higher risk in organizations and on the job, since their SOC levels are lower than the ones of male managers and middle to older aged adults in the workplaces.

## 7. Conclusions

This research focused on exploring SOC during the experience of COVID-19 in the workplace, responding to the question of what kind of SOC level managers express during this time, through the questionnaire survey and the interviews conducted. These findings can provide insight into the present situation at work and also anticipate future directions which are concluded in the following and highlighted in the recommendations for theory and practice.

Findings show that managers score highest in comprehensibility, followed by manageability, and then meaningfulness. In general, male white Afrikaans-speaking managers score the highest in all sub-categories, as well as in total SOC scores with SOC-scores in the higher ranges. Female managers scored in the medium range in all sub-categories, as well as in the total SOC scores. In total SOC scores, women score a minimum of 15 scores lower (163) than the highest SOC score, which was scored by a male manager (178). Men working in future-orientated fields, such as Engineering and IT score higher in comprehensibility and manageability and total SOC scores than men in other professional positions (e.g., HR or Finances, etc.). It can also be summarized that male managers between 47 and 57 score higher than younger men and women. The lowest SOC scores in comprehensibility are scored by two women (37 and 34 years old), while the highest score in meaningfulness is reached by a white Afrikaans speaking person aged 49 and the lowest score is reached by a 63-year-old white Afrikaans speaking male manager.

It might be assumed that age plays a role in the SOC scores in terms of comprehensibility and manageability, since SOC might be related to understanding complexities of the workplace and an ability to anticipate future directions which might grow with age and experience on the job and in the specific field of work. Further, comprehensibility might also relate to the analytical thought styles and manageability of the resources that are available, or which can be activated or organized. Meaningfulness seems to be rather dependent on the ascription to meaning in life and at work and might, in the case of the lowest scorer, be impacted by age and other factors, such as belief system or family background.

Interviews show that comprehensibility in the workplace during COVID-19 and the anticipation of future workplaces and rapid changes are connected to topics and understanding of (1) workplace and job changes; (2) increasing use of technologies; (3) remote work experiences, and (4) health and well-being. Manageability is associated with topics, such as (1) Skills and knowledge to manage; and (2) Managing resources. The mangers who score on the lower range mention upskilling and increasing knowledge and skills as an important strategy to increase manageability. Meaningfulness on the job is interlinked with managers’ ideas of (1) meaningfulness of work and career. It is connected to bringing solutions to major human challenges, good human-technological interaction which creates increased knowledge, face-to-face contact and human-human interaction which creates growth and brings psychological services, solving problems, having personal contact with people, having to solve challenges, achieving goals, creating a sustainable organization and having a job that is in itself meaningful. Managers do not relate their meaningfulness to their faith, religion or belief system, but rather to the improvement of human and technological cooperation, problem-solving and solution finding.

Managers within the data hardly refer explicitly to the COVID-19 situation, but rather refer to their anticipation of the Fourth Industrial Revolution and the creation of sustainable future workplaces.

Table 3 provides an overview of scores of SOC and the topics connected. Findings further show that the most complex responses are presented in the sub-component of comprehensibility, followed by manageability and meaningfulness. This correlates with the SOC scores being the highest in comprehensibility, manageability and meaningfulness. This means that the managers have a high understanding and knowledge of their work situation and the future implications, but to a lesser degree the ability to manage the situation and the resources, and to an even lesser degree they experience meaningfulness. This could be related to the COVID-19 situation which might be to a high degree comprehensible for most of the managers, but less manageable. It might also mean that the meaningfulness within this strongly impacting pandemic situation is decreased, or to a certain degree less experienced. Overall, however, the managers have a relatively strong SOC during the pandemic which leads to the interpretation that they are quite resilient and that they, despite the extreme challenging situation of COVID-19, remote work and the lockdown situation, have a strong life-orientation in general which comes into play, and which is kept up even during the challenging or even traumatic situation of the pandemic.

For future research the following is recommended: SOC scores should be explored not only in managers dealing with the pandemic within South Africa, but across different cultural contexts and industries. Further research needs to be carried out with regard to managerial SOC and gender, age and industry. Additionally, the South African sample is biased in terms of cultural belonging and first language group membership (English and Afrikaans), and future research needs to diversify the sample within the South African context.

On a practical note, organizations should explore their SOC of managers and how they cope with the COVID-19 situation. They should be aware that there might be differences in SOC scores and the ability to understand, manage and ascribe meaning to the situation and future work situations in terms of gender, age, position and background of the managers involved in leading the organization. Further, they should offer increasingly communication, training and resources to increase the SOC in managers and support them in managing present and future work situations. It might be of importance to support women leaders in a particular way, especially in male-dominated work environments. The same is true for colleagues under 40 years who might not have a broad and in-depth work experience and who might be impacted negatively (in terms of non-comprehensibility, non-manageability and decreased meaningfulness due to COVID-19) within their work context. Organizations can increase resilience by fostering understanding through communication, knowledge-based information, and an exchange of ideas and discussions on the anticipation of future directions. They might increase manageability by focusing on resources and resource distribution to support managers, and finally, but probably most important, they should focus on creating meaning at work for managers. Meaning might be an underestimated aspect which should be taken into consideration by organizations, and awareness around the topic should be fostered.

## Figures and Tables

**Table 1:** Overview of Research Methodology.

Research Design	Research Paradigm	Research Question	Research Objective
Mixed method using qualitative and quantitative methods (interview and questionnaire)	Phenomenological hermeneutical research paradigm, analysing the data from a qualitative perspective	What is the sense of coherence of managers during Covid-19 in the new world of work of the Fourth Industrial Revolution	The aim of this study was to explore the SOC in managers across different organisations in South Africa during COVID-19, aiming at understanding the SOC levels of managers in-depth and holistically and their sub-components in the context of their present and future of work situation.

**Table 2:** Biographical Information of Sample.

Participant	Gender	Age	Race	First Language	Industry	Position in the Company
1	Male	57	White	English	Manufacturing	Managing Director
2	Male	51	White	Afrikaans	Electronic manufacturing	Senior Executive Product Development
3	Male	27	White	Afrikaans	Psychiatry	Senior Research Officer
4	Male	51	White	Afrikaans	Procurement, Wholesale & Human Resource	Financial Director
5	Female	52	White	Afrikaans	Medical	Embryologist 1991–2019 Medical Sales Representative
6	Female	37	White	Afrikaans	IT/Telecommunication	Executive/Shareholder
7	Female	57	White	Afrikaans	Finance	HR Manager
8	Female	58	White	English	Retail	Senior Bookkeeper & Head of Accountant
9	Female	38	White	English	Professional Services	Executive Head
10	Male	47	White	Afrikaans	IT/Software Development	Executive
11	Male	51	White	Afrikaans	Construction	Commercial Director
12	Female	34	White	English	Previous–academia; current–performance coaching	Performance coach
13	Male	41	White	Afrikaans	Utilities	Deputy Engineering Manager
14	Male	63	White	English	Old Age Home	Executive Director
15	Female	26	Coloured	English	Assisted Reproduction/Embryology/Obstetrics and Gynecology	Assistant Research Officer
16	Male	52	White	English	Engineering	Director
17	Male	49	White	Afrikaans	Financial Services (retirement funds)	CEO

**Table 3:** Overview of SOC Findings.

SOC Scores	Comprehensibility	Manageability	Meaning-Fulness	Overall SOC
Highest	P1 (67)	P1 and P4 (63)	P17 (55)	
2 highest	P10 (61)	P2 (62)	P4 (52)	
3 Highest	P4 and P11 (60)	P10(61)	P10 (49)	
Lowest	P6 (37)	P12 (34)	P14 (36)	
Overall Highest				P1 (178) P4 (176) P10 (171) P17 (169) P11 and P13 (161) P2 (155) P8 (153) P3 and P9 (152)
Overall lowest				P12 (124)
Qualitative insights	1. workplace and job changes; 2. increasing use of technologies; 3. remote work experiences, and 4. health and well-being.	1. Skills and knowledge to manage; and 2. Managing resources.	1. meaning-fulness of work and career.	

## Data Availability

Data are available and can be provided if need be.
